# Relationship between Erythrocyte Fatty Acid Composition and Psychopathology in the Vienna Omega-3 Study

**DOI:** 10.1371/journal.pone.0151417

**Published:** 2016-03-10

**Authors:** Sung-Wan Kim, Min Jhon, Jae-Min Kim, Stefan Smesny, Simon Rice, Michael Berk, Claudia M. Klier, Patrick D. McGorry, Miriam R. Schäfer, G. Paul Amminger

**Affiliations:** 1 Department of Psychiatry, Chonnam National University Medical School, Gwangju, Republic of Korea; 2 Department of Psychiatry, University Hospital Jena, Jena, Germany; 3 Orygen, The National Centre of Excellence in Youth Mental Health, Centre for Youth Mental Health, The University of Melbourne, Parkville, Victoria 3052, Australia; 4 IMPACT Strategic Research Centre, School of Medicine, Deakin University, Geelong, Australia; 5 Department of Child and Adolescent Medicine, Medical University of Vienna, Vienna, Austria; Institute for Health & the Environment, UNITED STATES

## Abstract

This study investigated the relationship between erythrocyte membrane fatty acid (FA) levels and the severity of symptoms of individuals at ultra-high risk (UHR) for psychosis. Subjects of the present study consisted of 80 neuroleptic-naïve UHR patients. Partial correlation coefficients were calculated between baseline erythrocyte membrane FA levels, measured by gas chromatography, and scores on the Positive and Negative Syndrome Scale (PANSS), Global Assessment of Functioning Scale, and Montgomery–Asberg Depression Rating Scale (MADRS) after controlling for age, sex, smoking and cannabis use. Subjects were divided into three groups according to the predominance of positive or negative symptoms based on PANSS subscale scores; membrane FA levels in the three groups were then compared. More severe negative symptoms measured by PANSS were negatively correlated with two saturated FAs (myristic and margaric acids), one ω-9 monounsaturated FA (MUFA; nervonic acid), and one ω-3 polyunsaturated FA (PUFA; docosapentaenoic acid), and were positively correlated with two ω-9 MUFAs (eicosenoic and erucic acids) and two ω-6 PUFAs (γ-linolenic and docosadienoic acids). More severe positive symptoms measured by PANSS were correlated only with nervonic acid. No associations were observed between FAs and MADRS scores. In subjects with predominant negative symptoms, the sum of the ω-9 MUFAs and the ω-6:ω-3 FA ratio were both significantly higher than in those with predominant positive symptoms, whereas the sum of ω-3 PUFAs was significantly lower. In conclusion, abnormalities in FA metabolism may contribute to the neurobiology of psychopathology in UHR individuals. In particular, membrane FA alterations may play a role in negative symptoms, which are primary psychopathological manifestations of schizophrenia-related disability.

## Introduction

Schizophrenia is a severe mental illness that typically begins in adolescence or early adult life. It is a heterogeneous disorder with diverse symptoms which are generally characterised as positive or negative [1,2]. Positive symptoms, such as delusions and hallucinations, are considered to be more transient; negative symptoms, such as blunting of affect and passive withdrawal, are regarded as more persistent and contribute more to impairment [3].

The dopamine hypothesis is one of the most influential theories about the etiology of schizophrenia. Initially, the hypothesis emphasised an etiologic role of hyperdopaminergia, but this was subsequently reconceptualised to specify subcortical hyperdopaminergia with prefrontal hypodopaminergia [4]. Normally, the prefrontal dopamine system suppresses the limbic dopamine system; however, in schizophrenia, this suppression seems to be reduced due to disturbed prefrontal dopaminergic activity, leading to elevated limbic dopaminergic activity [5]. The dopamine hypothesis is concordant with both the negative symptoms of schizophrenia, likely linked with prefrontal hypodopaminergia, and the positive symptoms, strongly related to enhanced limbic dopaminergic activity [6].

While psychopathology is traditionally explained by disturbed neurotransmitter function, polyunsaturated fatty acids (PUFAs) gained interest in terms of the etiology of structural and functional abnormalities of the developing nervous system in schizophrenia. PUFAs are major constituents of neuronal and myelin membranes but have also important functions in the regulation of neuronal migration, pruning, and synaptic plasticity. Evidence from animal studies shows that a profound experimental ω-3 PUFA deficiency can alter dopaminergic and serotonergic neurotransmitter systems. These associations between PUFA status and neurotransmission may explain the role of PUFAs on human brain function and behaviour [7]. PUFAs are also selectively concentrated in synaptic neuronal membranes and regulate vascular and immune functions that affect the central nervous system [8]. The ω-3 PUFAs have anti-inflammatory effects, suppress interleukin-1β, tumor necrosis factor-α and interleukin-6, whereas ω-6 PUFAs do not [9]. A positive correlation between ω-6 PUFAs and intracellular phospholipase-2 (inPLA_2_) activity was observed in patients at ultra-high risk (UHR) for psychosis, while supplementation with ω-3 PUFA resulted in a significant decrease in inPLA_2_ activity [10]. These potential pro-inflammatory effects of ω-6 PUFAs and anti-inflammatory effects of ω-3 PUFAs may be associated with psychopathology in different way.

The accumulated evidence suggests that patients with schizophrenia are deficient in key PUFAs [11]. However, it is not clearly understood which PUFAs are altered and how PUFA deficiencies relate to psychotic symptoms. Several studies have demonstrated an association between PUFA deficiencies and the severity of the negative symptoms of schizophrenia, whereas others have reported inconsistent findings [12–14]. It is also not known if PUFA alterations reflect risk factors such as nutrition, are a result of a biological predisposition to schizophrenia or if they are associated only with an existing psychotic state, where they might be influenced by disease related pathways such as oxidative damage to lipids [14]. As antipsychotic medication and also the progression of illness may alter PUFA levels, FA research focussed on drug-free first-episode patients to minimise this confound. Evaluations of PUFA profiles in individuals at the prodromal (UHR) stage of schizophrenia will help even more to clarify the role of FAs in schizophrenia etiopathogenesis.

Recently, α-linolenic acid (ALA), eicosapentaenoic acid (EPA), and ω-6 PUFAs were found to be significantly lower in young UHR individuals than in healthy controls [15]. This suggests that FA deficiency may be present prior to illness onset and may be useful as a potential risk biomarker for the high-risk state, using the classification of Davis [16]. To investigate state marker properties, the present exploratory study aimed to investigate the relationship between erythrocyte membrane FA levels and the severity of symptoms in individuals at UHR for psychosis. The association between membrane FAs and psychotic symptoms was also evaluated according to the predominance of positive or negative symptoms.

## Methods

### Subjects

The study sample consisted of 81 antipsychotic-naïve UHR individuals aged 13–25 years involved in a randomised controlled trial of ω-3 FA supplementation (clinicaltrials.gov identifier: NCT00396643) [17]. Participants were recruited from the psychosis detection unit at the Medical University of Vienna, Austria. Levels of erythrocyte membrane FAs were determined in 80 participants at baseline. All study subjects met one or more of the three operationally defined and validated UHR criteria: attenuated positive psychotic symptoms, transient psychosis, and genetic risk plus a decrease in functioning [18]. These criteria comprise a combination of trait and state factors that identify people whose risk of becoming psychotic may approach 40% within a 12-month period [19,20]. The presence of attenuated psychotic symptoms and transient psychosis were determined in a semi-structured interview applying Positive and Negative Syndrome Scale (PANSS) [21] cut off scores for symptom severity proposed by Morrison et al [22] and frequency and duration criteria by Yung et al [19]. Genetic risk was composed of individuals who had a schizotypal personality disorder or a family history of psychotic disorder in a first-degree relative (as assessed with the Family History Research Diagnostic Criteria) [23] and a decrease of functioning of 30% or more on the Global Assessment of Functioning (GAF) Scale [24] within the past year. Exclusion criteria included a history of a previous psychotic disorder or manic episode, substance-induced psychotic disorder, acute suicidal or aggressive behaviour, current DSM-IV [24] diagnosis of substance dependence (except cannabis dependence), neurological disorders, or IQ less than 70. People with gross structural brain abnormalities observable on their magnetic resonance image (MRI) scan were excluded. Also excluded were people who had previous treatment with an antipsychotic or mood-stabilizing agent for more than one week and people who had taken omega-3 PUFA supplements within 8 weeks of being included in the trial. Finally, people showing laboratory values more than 10% outside the normal range for transaminases, thyroid hormones, C-reactive protein, or bleeding parameters were excluded.

The study was carried out according to the latest version of the Declaration of Helsinki and was approved by the Medical University of Vienna Ethics Committee. Written informed consent was obtained from all participants (written consent of parent or guardian was obtained for those aged < 18 years). [25]

### Analysis of erythrocyte membrane FA composition

Erythrocyte membrane phospholipid composition closely reflects that of neuronal membranes and provides an easily accessible indicator of brain phospholipids [26,27]. We separated plasma and erythrocytes from whole blood samples and analysed the FA composition in the phospholipids of the phosphatidylethanolamine (PE) fraction of erythrocyte membranes. Fatty acid containing erythrocyte membrane extracts were evaporated, and phospholipid fractions were dissolved using thin-layer gas chromatography (50 ml chloroform, 37.5 ml methanol, 3.5 ml glacial acetic acid, and 2 ml distilled water). Phospholipid fractions were then saponified and esterified [28–30].

The PE fraction, which contains mono- and polyunsaturated fatty acids, is situated largely on the inner side of membranes. The presumed pathology underlying phospholipid alterations in psychotic illness includes increased peroxidation (i.e., oxidative stress) leading to increased oxidative damage of PUFA, all related to ongoing excitotoxicity. This pathology particularly affects the cytosolic (inner) side of cell membranes. As PE is the most common phospholipid on the inner side of membranes in the brain, the FA levels (in mol% of total fatty acid levels) of this PE fraction were included in our statistical analysis. Additional analysis details are presented in a previous study [10].

Using gas chromatography and standard quantitation methods, values for the following classes of FAs were obtained: saturated FAs (14:0, 16:0, 17:0, 18:0); monounsaturated (MU) FAs (18:1ω-9, 20:1ω-9, 20:3ω-9, 22:1ω-9, 24:1ω-9), ω-6 PUFAs (18:2ω-6, 18:3ω-6, 20:3ω-6, 20:4ω-6, 22:2ω-6, 22:4ω-6), and ω-3 PUFAs (18:3ω-3, 20:5ω-3, 22:5ω-3, 22:6ω-3).

### Psychiatric outcome measures

Psychotic symptom severity was assessed using the PANSS, which has been widely used to measure psychotic symptoms with good validity and reliability [21,31,32]. The PANSS consists of three subscales (positive, negative, and general psychopathology) and is used to classify patients with schizophrenia as dominant positive symptoms, dominant negative symptoms, or mixed type. Subjects were divided into three groups according to the predominance of positive or negative symptoms, which was determined based on PANSS positive and negative subscale scores. Positive symptom predominance was defined as a score of 2 or higher on the positive subscale relative to the negative subscale. Negative symptom predominance was defined as a score of 2 or higher on the negative subscale relative to the positive subscale. Mixed type was defined as: -1 ≤ positive minus negative subscale score ≤ 1.

The GAF scale was used to measure social, occupational, and psychological functioning [24,33]. The GAF is an analogue scale (0–100), with higher scores indicating better functioning. The Montgomery–Asberg Depression Rating Scale (MADRS) was used to assess depressive symptoms [34]. Subjects were evaluated by experienced clinicians (raters) who were trained to administer these tools. Inter-rater reliability estimates were high (intra-class correlation coefficients > 0.92). Cronbach’s alpha values for the PANSS and MADRS in this study were 0.849 and 0.885, respectively.

### Statistical analysis

Partial correlation coefficients were calculated between membrane FA levels and scores on the PANSS (total score and scores on the three subscales), MADRS, and GAF after controlling for age, sex, smoking and cannabis use. Levels of FAs including the sum of MUFAs, ω-6 PUFAs, and ω-3 PUFAs; and the ω-6:ω-3 FA ratios were compared among the three PANSS subgroups (dominant positive symptoms, dominant negative symptoms, and mixed type) using analysis of variance (ANOVA) followed by post hoc Bonferroni’s tests. All statistical tests were two-tailed, with a significance level of P<0.05.

## Results

Partial correlations between erythrocyte membrane PE fatty acids and symptom severity measures after controlling for age, sex, smoking, cannabis use, and scores on MADRS and GAF are presented in Table 1. The ω-6:ω-3 FA ratio was positively correlated with PANSS total and negative symptom scores and sum of the ω 3 fatty acids was negatively associated with PANSS negative symptom scores. Total PANSS scores were negatively correlated with two saturated FAs (myristic and margaric acids) and one ω-9 MUFA (nervonic acid) and positively correlated only with two ω-9 MUFAs (eicosenoic and erucic acids) and two ω-6 PUFAs (γ-linolenic and docosadienoic acids). PANSS positive symptom scores were significantly correlated with nervonic acid. In terms of PANSS negative symptom and general psychopathology scores, negative correlations were found with two saturated FAs (myristic and margaric acids) and one ω-9 MUFA (nervonic acid), whereas positive correlations were found with two ω-9 MUFAs (eicosenoic and erucic acids) and two ω-6 PUFAs (γ-linolenic and docosadienoic acids). One ω-3 PUFA (docosapentaenoic acid, (DPA)) was negatively correlated with PANSS negative symptoms scores. GAF scores were correlated with one ω-6 PUFA (docosadienoic acid).

**Table 1:** Partial correlations coefficients between erythrocyte membrane phosphatidylethanolamine lipids and psychiatric measures controlling for age, sex, smoking, and cannabis use.

	PANSS				MADRS	GAF
	Total	Positive	Negative	General		
Myristic acid (14:0)	-.305**	-.085	-.301**	-.297**	-.177	.176
Palmitic acid (16:0)	-.167	-.082	-.196	-.120	-.027	-.017
Margaric acid (17:0)	-.260*	.058	-.333**	-.249*	.051	.069
Stearic acid (18:0)	.085	-.083	.101	.119	.008	-.085
Oleic acid (18:1n-9)	-.029	.047	.030	.010	.106	.023
Eicosenoic acid (20:1n-9)	.378***	.128	.478***	.268*	-.004	-.193
Mead acid (20:3n-9)	.198	.052	.205	.187	.124	-.043
Erucic acid (22:1n-9)	.395***	.158	.446***	.314**	.072	-.182
Nervonic acid (24:1n-9)	-.339**	-.231*	-.277*	-.316**	-.185	.108
Linoleic acid (18:2n-6)	-.019	-.063	.006	-.012	-.012	.044
γ-Linolenic acid (18:3n-6)	.332**	.109	.383***	.268*	.160	-.139
Dihomo-γ-linolenic acid (20:3n-6)	-.029	-.128	.081	-.066	-.108	.202
Arachidonic acid (20:4n-6)	-.030	.046	-.036	-.049	-.080	.073
Docosadienoic acid (22:2n-6)	.388***	.118	.428***	.336**	.096	-.247*
Adrenic acid (22:4n-6)	.104	.104	.108	.061	-.088	-.082
α-Linolenic acid (18:3n-3)	-.074	-.131	-.079	-.015	-.157	.126
Eicosapentaenoic acid (20:5n-3)	-.085	.004	-.149	-.040	.144	.039
Docosapentaenoic acid (22:5n-3)	-.215	-.052	-.267*	-.165	-.088	.199
Docosahexaenoic acid (22:6n-3)	-.075	.072	-.173	-.031	.058	.070
Sum of monounsaturated fatty acids	.184	.098	.220	.125	.100	-.051
Sum of polyunsaturated fatty acids	-.028	.031	-.035	-.039	-.078	.103
Sum of n-6 fatty acids	.022	.028	.051	-.013	-.102	.069
Sum of n-3 fatty acids	-.146	.021	-.239*	-.089	.017	.133
n-6 to n-3 fatty acids ratio	.232*	.057	.305**	.165	.052	-.189

* p-value < 0.05

** p-value < 0.01

*** p-value < 0.001

PANSS, Positive and Negative Syndrome Scale; MADRS, Montgomery–Asberg Depression Rating Scale’ GAF, Global Assessment of Functioning

Demographic and clinical characteristics of subgroups according to PANSS scores are shown in Table 2. No significant differences were found with regard to age; sex; use of alcohol, cannabis, tobacco, or any illicit drug; MADRS scores; or transition to psychosis. GAF scores were significantly lower in the negative-symptoms-dominant group than in the positive-symptoms-dominant group (p-value = 0.004, ANOVA post-hoc analysis).

**Table 2:** Comparison of demographic and clinical characteristics of participants according to dominance of positive or negative symptoms.

	Total	Subtypes			
	(n = 80)	Positive dominant[a] (N = 38)	Mixed[b] (N = 23)	Negative dominant[c] (N = 19)	*p* value
Sex, female, n	54 (67.5)	26 (68.4)	16 (69.6)	12 (60.0)	0.764
Age, years, mean (SD)	16.5 (2.1)	16.6 (2.2)	16.6 (2.7)	16.1 (1.3)	0.651
Alcohol					0.949
Less than weekly	45 (56.3)	22 (57.9)	12 (52.2)	12 (60.0)	
1–6 drinks per week	21 (26.3)	10 (26.3)	7 (30.4)	4 (20.0)	
Daily	14 (17.5)	6 (15.8)	4 (17.4)	4 (20.0)	
Cannabis					0.410
No	68 (85.0)	33 (93.5)	19 (82.6)	17 (85.0)	
≤ 2g per week	8 (10.0)	2 (6.5)	4 (17.4)	2 (10.0)	
> 2g per week	4 (5.0)	3 (0.0)	0 (7.7)	1 (5.0)	
Smoking, yes, n (%)	42 (52.5)	17 (45.2)	12 (50.0)	13 (65.2)	0.340
Any illicit drug, n (%)	14 (17.5)	6 (9.7)	4 (15.4)	4 (30.4)	0.922
Transition, yes, n (%)	13 (16.3)	4 (10.5)	4 (17.4)	5 (25.0)	0.353
MADRS, score, mean (SD)	18.2 (8.8)	16.0 (8.2)	20.9 (9.8)	19.2 (7.9)	0.086
GAF, score mean (SD)	60.4 (12.5)	65.0 (11.4)	58.3 (13.4)	54.0 (10.5)	**0.003**^†^

^†^a > c, p-value = 0.004. post-hoc analysis.

MADRS, Montgomery–Asberg Depression Rating Scale’ GAF, Global Assessment of Functioning

Table 3 include the results of group comparison of FA levels among the three PANSS subgroups. The sum of ω-3 PUFAs and myristic acid levels were significantly lower in the negative-symptoms-dominant group than in the positive-symptoms-dominant group. The sum of the ω-9 MUFAs and two single ω-9 MUFAs (eicosenoic and erucic acids), two ω-6 PUFAs (γ-linolenic and docosadienoic acids), and the ω-6:ω-3 FA ratio were all significantly higher in the negative-symptoms-dominant as compared to the positive-symptoms-dominant group.

**Table 3:** Comparison of erythrocyte membrane phosphatidylethanolamine lipids levels according to dominance of positive or negative symptoms.

	Subtypes				
	Positive dominant[a] (N = 38)	Mixed[b] (N = 23)	Negative dominant[c] (N = 19)	*p* value	Post-hoc analysis(Bonferroni correction)
Myristic acid (14:0)	0.59 (0.21)	0.54 (0.16)	0.48 (0.15)	0.102	
Palmitic acid (16:0)	23.64 (3.16)	23.97 (2.04)	22.45 (2.41)	0.168	
Margaric acid (17:0)	2.50 (0.55)	2.27 (0.67)	1.94 (0.50)	**0.004**	a>c**
Stearic acid (18:0)	12.04 (2.36)	12.99 (2.52)	12.14 (1.85)	0.278	
Oleic acid (18:1n-9)	22.96 (2.58)	23.08 (2.56)	24.07 (3.57)	0.366	
Eicosenoic acid (20:1n-9)	0.76 (0.31)	0.92 (0.38)	1.24 (0.40)	**< 0.001**	a<c***, b<c*
Mead acid (20:3n-9)	0.11 (0.50)	0.12 (0.47)	0.13 (0.40)	0.313	
Erucic acid (22:1n-9)	0.65 (0.91)	1.04 (1.01)	1.38 (0.78)	**0.017**	a<c*
Nervonic acid (24:1n-9)	0.06 (0.02)	0.05 (0.02)	0.05 (0.15)	0.219	
Linoleic acid (18:2n-6)	6.26 (0.69)	5.91 (0.79)	6.69 (2.26)	0.142	
r-Linolenic acid (18:3n-6)	0.33 (0.13)	0.41 (0.16)	0.45 (0.14)	**0.008**	a<c**
Dihomo-γ-linolenic acid (20:3n-6)	1.61 (0.42)	1.62 (0.43)	1.70 (0.34)	0.722	
Arachidonic acid (20:4n-6)	15.95 (2.11)	14.95 (2.04)	15.35 (2.02)	0.180	
Docosadienoic acid (22:2n-6)	0.06 (0.05)	0.10 (0.04)	0.11 (0.04)	**<0.001**	a<c***, a<b*
Adrenic acid (22:4n-6)	4.69 (0.71)	4.61 (1.10)	4.78 (0.82)	0.824	
a-Linolenic acid (18:3n-3)	0.19 (0.05)	0.21 (0.08)	0.19 (0.43)	0.543	
Eicosapentaenoic acid (20:5n-3)	0.50 (0.17)	0.49 (0.17)	0.43 (0.13)	0.270	
Docosapentaenoic acid (22:5n-3)	2.25 (0.36)	2.18 (0.53)	2.02 (0.37)	0.150	
Docosahexaenoic acid (22:6n-3)	2.83 (0.80)	2.51 (0.73)	2.36 (0.50)	0.050	
Sum of monounsaturated fatty acids	26.45 (3.01)	27.13 (2.98)	28.78 (3.11)	**0.027**	a<c*
Sum of polyunsaturated fatty acids	34.79 (3.30)	33.10 (3.48)	34.22 (2.42)	0.138	
Sum of n-6 fatty acids	28.90 (2.72)	27.60 (2.80)	29.09 (2.19)	0.113	
Sum of n-3 fatty acids	5.77 (1.09)	5.39 (1.31)	5.00 (0.76)	**0.043**	a>c*
n-6 to n-3 fatty acids ratio	5.13 (0.80)	5.38 (1.23)	5.94 (1.00)	**0.018**	a<c*

* p-value < 0.05

** p-value < 0.01

*** p-value < 0.001

## Discussion

In this exploratory study of individuals at UHR for psychosis, the PE fraction of erythrocyte membrane FAs were associated with severity of psychopathology. Negative symptoms were negatively associated with saturated FAs (myristic and margaric acids), ω-3 PUFA (DPA), and nervonic acid but positively associated with several other ω-9 MUFAs (eicosenoic and erucic acids) and ω-6 PUFAs (γ-linolenic and docosadienoic acids). The sum of the ω-9 MUFAs and the ω-6:ω-3 FA ratio were both significantly higher, whereas the sum of the ω-3 PUFAs was significantly lower in subjects with predominant negative symptoms compared with those with predominant positive symptoms. No associations were observed between FAs and MADRS scores, suggesting that the associations between FAs and negative symptoms were not confounded by depression, a potential concern given their overlapping phenomenology. To the best of our knowledge, this is the first study conducted in neuroleptic-naïve UHR subjects that has demonstrated a relationship between membrane FA levels and the severity of symptomatology. The findings suggest that cell membrane lipid biology may be useful to assess during the onset phase of psychotic disorders and are consistent with the observation that supplementation with omega-3 PUFAs might be an effective and longer-term preventive treatment in this cohort of UHR patients [17,35].

Our findings of an association between membrane FA level and negative symptoms are comparable to earlier studies conducted in patients with schizophrenia. Glen and colleagues found that patients with predominantly negative symptoms exhibited lower levels of ω-3 PUFAs in red blood cells compared with those with persistently positive symptoms and controls [12]. Additionally, patients with low erythrocyte PUFA (sum of ω-3 and ω-6 FA levels) had more negative symptoms than those with high PUFA levels [13]. In a study in drug naïve-patients with schizophrenia, ω-3 PUFA deficiency was associated with negative symptoms [14]. Accordingly, it was demonstrated that supplementation with ω-3-rich fish oil might result in a significant improvement in negative but not in positive symptoms of patients with schizophrenia [36]. In a clinical trial of EPA supplementation in patients with schizophrenia, an increase in membrane ω-3 PUFAs was significantly correlated with change in PANSS negative symptom scores and total scores [37].

The prefrontal cortex plays an important role in higher brain functions. The basic activity of this brain region is considered to be the orchestration of thoughts and actions in accordance with internal goals [38]. It has also been implicated in planning complex cognitive behaviour, executive function, decision making, and moderating social behaviour [39]. It has been assumed that hypofunctioning of the cortical and prefrontal dopamine systems contributes to cognitive impairment and negative symptoms in patients with schizophrenia, such as diminished emotional expression and avolition [40]. The relationship between ω-3 PUFA deficiency and negative symptoms is concordant with the dopamine hypothesis of schizophrenia. In animal studies, deficiencies in ω-3 PUFAs have been found to cause reductions in levels of dopamine and D2 receptors in the frontal cortex [41,42] and a hypofunction of the prefrontal dopamine system, reflecting a correlate of negative symptoms and cognitive impairments [6]. In addition, ω-3 PUFA deficiency of rats affects dopaminergic neurotransmission and mRNA expression of D2 receptor genes in the frontal cortex [43–45].

Animal studies showed that oral intake of ω-3 FAs can increase and decrease the number of D2 receptors in the frontal lobe and striate body, respectively [46,47]. In a clinical trial of patients with first-episode schizophrenia, improvement of negative symptoms induced by ethyl-EPA supplementation was associated with metabolic brain changes, particularly increases in glutathione [48]. Glutathione is an antioxidant that protects dopaminergic neurons from oxidative and excitatory damage [49,50].

White matter changes including dysregulated myelination are presented in patients with schizophrenia [51]. Reduced levels of PUFAs in erythrocyte membranes may be also related to white matter integrity throughout various brain areas including the frontal lobe. An imaging study of patients with early psychosis found that more severe negative symptoms were associated with lower levels of integrity in the white matter associated with total PUFA or arachidonic acid (an ω-6 PUFA), whereas no significant associations were found with positive symptoms [52]. Furthermore, animal studies suggested that PUFAs are important for the development, structure, and functioning of neurons and glial cells [53] and for neurite outgrowth, exocytosis, and monoaminergic signal transmission [54]. Thus, PUFA deficiency may impair dopaminergic and glutamatergic neurotransmission in the frontal lobe, which is linked to the negative symptoms of schizophrenia [13]. These biochemical, imaging, pharmacological, and animal experimental studies support our findings.

In particular, nervonic acid, a major constituent of myelin membranes, helps to maintain white matter integrity [52,55]. In the present study, nervonic acid was negatively correlated with scores of the PANSS (total score and scores on the three subscales) in UHR individuals, which is consistent with earlier studies of patients with schizophrenia.

Myristic acid is an anchorage moiety, and plays a role in attaching proteins to lipid biomembranes. Linkage of myristic acid to proteins is called myristoylation. It is involved in key cellular functions including apoptosis, cellular differentiation and intracellular signalling. Myristic acid is vulnerable to redox damage and can be rendered immunogenic when oxidised [56].

In a previous study of patients with schizophrenia, Sethom observed a decrease in the levels of ω-6 (arachidonic acid) and ω-3 (DHA) PUFAs but an increase in the ω-6:ω-3 ratio [14]. The higher ω-6:ω-3 PUFA ratio may be indicative of a pro-inflammatory response [57,58]. However, a stronger inflammatory response may increase the production of free radicals and reduce PUFA levels [59]. Reduced anti-inflammatory activity may be implicated in the negative symptoms and cognitive impairment observed during the acute stages of schizophrenia episodes [60]. Our finding of a higher ω-6:ω-3 ratio related to negative symptoms is consistent with previous research.

Among ω-9 MUFAs, eicosenoic acid and erucic acid (its elongation product) was correlated with negative symptoms and were higher in the negative-symptoms-dominant group. Eicosenoic acid was recently reported as a key biomarker for schizophrenia in a large global metabolic profiling study [61]. Serum eicosenoic acid levels were significantly higher in patients with schizophrenia, prior to antipsychotic treatment than in healthy controls [61]. Elevated eicosenoic acid (together with other observed metabolic abnormalities) may reflect increased FA catabolism as an alternative energy source, resulting from insufficient mitochondrial glycolytic energy generation in the brain [62]. The present findings indicate that such metabolic abnormalities occur in UHR individuals prior to schizophrenia, particularly in relation to negative symptoms. Increased levels of eicosenoic and/or erucic acid have also been reported in the brains of rats with cerebral ischemia [63], in cognitively impaired elderly people [64], and in children with regressive autism [65]. These observations suggest that increases in ω-9 FAs may be associated with central nervous system dysfunction [66].

Negative symptoms generally begin prior to schizophrenia onset and are regarded as a more primary psychopathology [67]. Indeed, the disability of patients with schizophrenia is associated primarily with negative symptoms. The negative symptoms of schizophrenia are less responsive to antipsychotic treatment than the positive symptoms, and specific treatment for negative symptoms is not yet available. Thus, our findings of an association between membrane FAs and negative symptoms may help to improve our understanding of schizophrenia and aid in the development of treatments for UHR individuals or patients with schizophrenia. Further studies are needed to identify the specific types of symptoms that respond best to treatment with ω-3 FA supplements.

Several limitations should be considered when interpreting these data. First, the sample size was relatively small and there were a large number of fatty acids assessed; as such, these data need to be regarded as exploratory, as there is a risk of both type 1 and 2 errors. Since all consecutive referrals over a period of approximately 2 years were considered for inclusion the sample is representative for UHR patients [17]. It is not known if the associations between alterations in FAs and the severity of symptoms are attributed to primary factors (i.e. the neurobiology underlying schizophrenia spectrum disorders) or secondary factors (i.e. altered dietary patterns arising from altered disease-related behaviour). Use of a UHR cohort goes some way to reducing this confound.

Our findings suggest that abnormalities in FA metabolism may be associated with the neurobiological etiology of psychopathology in UHR individuals. Alterations in membrane FAs correlated with negative symptoms, which are primary psychopathological features of schizophrenia and are closely associated with the long-term outcome of schizophrenia spectrum disorder. Membrane FAs could serve as useful biological markers for early stages of schizophrenia; modulation of membrane FAs may provide a preventative intervention in subjects at UHR for schizophrenia.
